# Impact of Concomitant Spinal Canal Stenosis on Clinical Presentation of Adult Onset Degenerative Lumbar Spondylolisthesis: A Study Combining Clinical and Imaging Spectrum

**DOI:** 10.7759/cureus.19536

**Published:** 2021-11-13

**Authors:** Chiranjit De, Chinmay De

**Affiliations:** 1 Trauma and Orthopaedics, Sandwell & West Birmingham NHS Trust, Birmingham, GBR; 2 Trauma and Orthopaedics, Burdwan Medical College, Bardhaman, IND

**Keywords:** imaging findings, neurology, clinical presentation, spinal canal stenosis, degenerative spondylolisthesis

## Abstract

Aim

Degenerative lumbar spondylolisthesis (DSL) is one of the reasons behind adult-onset backache due to degenerative spinal pathology. Clinical manifestations of this can range from asymptomatic patients to widely variable clinical signs and symptoms. Spinal canal stenosis (SCS) is the most common associated degenerative condition in the MRI of DSL. Moreover, other associated degenerative conditions may contribute significantly towards the clinical presentation. We have tried to assess the impact of SCS on the clinical symptomatology and presentation of the DSL by correlating the clinical and imaging findings.

Methods

This single-center prospective observational study has analysed 48 patients who were symptomatic due to DSL. The data was collected over a period of 18 months from January 2015 to June 2016 by screening through the adult patients presenting at the orthopaedic or spinal clinics with features suggestive of degenerative lumbar spine disease. Particular inclusion and exclusion criteria were developed as a screening tool and selected patients underwent imaging investigations. Patients had lumbar spine radiographs, both standing and flexion-extension view, and MRI of the lumbar spine. The presenting clinical features were documented. Their clinical and neurological assessment was done thoroughly by two qualified clinicians independently.

Results

The study population included 29 female (60.5%) and 19 male (39.5%) patients. The mean age of the study population was 49.5 years (SD 9.2 years). As per the radiological diagnostic criteria, 28 patients (58.3%) had features of SCS together with DSL and the rest of the 20 patients (41.7%) had DSL without SCS. Axial back pain and claudication had a statistically significant association with imaging findings. Similarly, patients with associated canal stenosis had statistically significant sensory and motor deficits, altered deep tendon reflexes. Facet joint angle more than 45 degrees at the level of the slip had a higher incidence of indicative presenting symptoms. However, this was not statistically proven.

Conclusion

DSL is a heterogeneous condition with the simultaneous presence of different degenerative processes in the lumbar spine at various stages. Hence, clinical presentations are widely variable. The concomitant presence of SCS significantly influences the clinical symptomatology with correlation to the MRI findings. Therefore, a judicious weighing of the clinical and imaging findings is crucial for prudent management planning for cases of DSL.

## Introduction

Degenerative lumbar spondylolisthesis (DSL) is one of the reasons behind adult-onset backache due to degenerative spinal pathology. In DSL, the cranial vertebra slips over the caudal vertebra due to degenerative changes in the lumbar spine. Unlike spondylolytic spondylolisthesis, there is no pars interarticularis defect. Moreover, there is no dissociation between the neural arch and the vertebral body of the slipping segment. This condition can be asymptomatic in a group of patients [1]. However, patients may present with various symptoms according to the stage of the disease itself and because of the other associated degenerative changes in the spine. Symptoms of degenerative listhesis are more due to rotatory deformity rather than a simple anteroposterior displacement [2]. Therefore, very limited information can be understood from a plain radiograph making MRI the gold standard investigation for this condition [2].

Major degenerative changes that might be associated with the slip could be disc degeneration leading to segmental instability [3], facet joint arthritis with loss of structural integrity, laxity of the posterior stabilizing ligaments, and non-effective muscular stabilization effect [4]. Moreover, vertebral translation with ligamentum flavum hypertrophy can cause spinal canal stenosis (SCS) and this is one of the most commonly reported concomitant findings in the MRI [1]. Further progression may cause neuroforaminal stenosis leading to various clinical manifestations.

Due to the natural progression of the disease, the clinical manifestation of DSL can range from asymptomatic patients to widely variable clinical signs and symptoms. Moreover, other associated degenerative conditions may contribute significantly towards the clinical presentation. Patients may present with various combinations of axial back pain, radiculopathy, neurogenic claudication, different degrees of motor and sensory loss, and various neurologic findings [5]. As SCS has been reported in the literature to be the most common associated degenerative condition in MRI, we have tried to assess the impact of this condition on the clinical symptomatology of the DSL. We have clinically assessed adult symptomatic patients presenting with DSL and have tried to find any relation with the MRI with regards to the findings of SCS. The results of this study can act as a symptomatology predictor for adult-onset DSL.

## Materials and methods

This single-centre prospective observational study has analysed 48 patients who were symptomatic due to DSL. The Clinical Research Ethics Committee, Burdwan Medical College, approved this project (Approval No: BMC/PG/277/1(1)). The data was collected over a period of 18 months from January 2015 to June 2016 by screening the adult patients presenting at the orthopaedic or spinal clinics with features suggestive of degenerative lumbar spine disease. As the number of patients attending these clinics with degenerative symptomatic lumbar spine disease was significantly high, particular inclusion and exclusion criteria were developed as a screening tool. The criteria have been explained in Table 1. This screening tool was an integral part of the study design as it identified the suspected cohort and helped in the judicious use of the valuable diagnostic resources.

**Table 1:** Inclusion and exclusion criteria used as screening tool for the study

INCLUSION CRITERIA
Patient age more than 35 years
Clinically suffering from degenerative lumbar spine disease
Imaging evidence of degenerative lumbar spondylolisthesis
EXCLUSION CRITERIA
Traumatic lumbar spine pathology
Metastatic disease of spine, especially lumbar region
Previous neuro-vascular pathology of both/either of the limbs
Associated lower limb fractures
Patients with head injuries and/or its residual morbidities
Patients having myopathies affecting lower extremities
Previous surgery in the dorso-lumbar region

All screened suspected patients underwent basic investigation in the form of lumbar spine radiograph both in anteroposterior and lateral view. If this detected DSL, they were included in the study and were investigated further as per the study protocol. Their clinical assessment was done thoroughly by two qualified clinicians independently and the final clinical findings were documented when they both reached a unanimous decision.

The first tier of clinical assessment was based on symptoms such as the presence of axial back pain, radiculopathy, and neurogenic claudication. Back pain was the most common and consistent presentation among the patients and there have been co-incidence with referred and radicular distal extremity pain. Therefore, we have also assessed patients with respect to the compound presentation of back pain with variable distal extremity pain. The next tier of clinical assessment was based on signs of sensory deficit, motor weakness, the Straight Leg Raise Test (SLRT), and alteration of deep tendon reflexes. The sensory assessment was done for bilateral lower limb dermatomes from L1 to L5 and S1. Power assessment was done in the key group of muscles such as hip flexors (L2), knee extensors (L3), ankle dorsiflexors (L4), long toe extensors/Extensor hallucis longus (L5), and ankle plantar flexors (S1). Sensory and motor weakness were expressed as categorical data. If there were neurologic deficits in any dermatomes or myotomes, this was classed as ‘Present’ for interpretation of the results. For interpreting SLRT, 45 degrees was considered as the cut-off for considering this test as ‘positive’ or ‘negative’ for sciatic nerve stretch signs for categorical interpretation. Similarly, patients were assessed for ankle and knee jerk. If any of the reflexes were found altered, they were categorised as ‘impaired’ for interpretation of the data.

All the patients included in the study had baseline lumbar spine radiographs. For confirmation of the diagnosis, some of the patients with low-grade spondylolisthesis underwent dynamic imaging with flexion and extension lateral radiograph of the lumbar spine to unmask the pathology. Anteroposterior-oblique views were obtained for some of the patients to rule out any evidence of pars-interarticularis lysis. MRI has been the most effective tool for radiological assessment. As MRI is the gold standard investigation for such conditions, it provided the maximum information for the degenerative condition and other associated contributory conditions. We have used a 1.5 T magnetic resonance system with standard lumbosacral imaging protocol using a surface coil. For interpretation of the imaging findings, both T1 and T2 weighted images with axial, coronal, and sagittal section sequences were used and interpretation was done by a senior musculoskeletal radiologist experienced in such reporting. We have particularly focused on the presence of SCS in conjunction with DSL considering the objective of this study. Anteroposterior canal diameter in the midline less than 10 mm or cross-sectional diameter <100 mm^2^ were considered as radiological diagnostic criteria for categorising the cases as SCS based on the MRI [6]. Therefore, all the patients with DSL included in the study were categorised based on the ‘presence’ or ‘absence’ of SCS in the MRI. We have analysed the clinical symptomatology with respect to the presence of SCS among patients suffering from DSL. Additionally, we have assessed the facet joint angle of the symptomatic patients and tried to interpret the relationship with the clinical presentation.

Statistical analysis was done by expressing the continuous variables as mean ± standard deviation (SD) and compared using Student’s t-test. Categorical variables were expressed with respect to the presence of specific clinical features and radiological findings. They were expressed as percentages and compared using the chi-square test. All statistical tests of significance were two-tailed, and p values < 0.05 were considered statistically significant. Statistical analyses were performed using SPSS for Windows, Version 16 (Released 2007; SPSS Inc., Chicago).

## Results

After screening the adult patients presenting with features of degenerative lumbar spine disease, a total of 48 patients with DSL were included in the study. The study population included 29 female (60.5%) and 19 male (39.5%) patients. The mean age of the whole study population was 49.5 years with an SD of 9.2 years. As per the radiological diagnostic criteria, 28 patients (58.3%) had features of spinal canal stenosis together with DSL, and the rest of the 20 patients (41.7%) had DSL without spinal canal stenosis.

Table 2 shows the relation between the clinical presentation and the MRI findings among the study population. There is a statistically significant association between the DSL with axial back pain (p-value 0.001) and claudication (p-value 0.002) in the presence of SCS altogether. However, symptoms of radiculopathy (p-value 0.241) had no statistically significant relationship with the concomitant presence of SCS with degenerative listhesis.

**Table 2:** Clinical presentation and MRI findings for patients with DSL DSL: degenerative spondylolisthesis; SCS: spinal canal stenosis

Clinical Findings		MRI Findings		p-value
		DSL without SCS (N=20)	DSL with SCS (N=28)	
Axial Back Pain	Present	16 (80%)	8 (28.6%)	0.001
	Absent	4 (20%)	20 (71.4%)	
Radiculopathy	Present	8 (40%)	16 (57.1%)	0.241
	Absent	12 (60%)	12 (42.9%)	
Claudication	Present	2 (10%)	18 (64.3%)	0.002
	Absent	18 (90%)	10 (35.7%)	

Further assessment of the backache with the degree of distal extremity pain was done. Those having distal extremity pain were sub-classified into two groups: the first group with referred pain to the anterior thigh without radiating distal to the knee and the second group with pain radiating distally to the knee joint. Table 3 describes the distribution of the patients with respect to the backache with distal extremity pain characteristics and MRI findings. There has been no characteristic pattern with or without associated SCS.

**Table 3:** Backache with distal extremity pain and MRI findings for patients with DSL DSL: degenerative spondylolisthesis; SCS: spinal canal stenosis

MRI Findings	Backache with Distal Extremity Pain		
	Absent	Present	
	No referred /radiating pain	Referred to the thigh	Radiating distal to the knee
				DSL without SCS	16/20 (80%)	0/20 (0%)	4/20 (20%)
DSL with SCS	8/28 (28.6%)	8/28 (28.6%)	12/28(42.8%)				
				Total	24/48(50%)	8/48 (16.6%)	16/48 (33.4%)

Table 4 summarises different clinical examination findings among the study population. Sensory deficit (p-value 0.01) and altered motor power (p-value 0.042) had a statistically significant relation with the concomitant presence of SCS with degenerative spondylolisthesis. To reiterate, sensory and motor weakness were expressed as categorical data. If there was a neurologic deficit in any dermatomes or myotomes, this was classed as ‘Present’ and ‘Reduced’ for interpretation of the results respectively. However, SLRT (p-value 0.364) had no statistically significant relationship with the MRI findings with the presence of SCS. On the other hand, patients with SCS with DSL had statistically significant (p-value 0.002) findings of altered deep tendon reflexes in the lower extremity.

**Table 4:** Clinical findings and MRI findings for patients with DSL DSL: degenerative spondylolisthesis; SCS: spinal canal stenosis

Clinical Findings		MRI Findings		p-value
		DSL without SCS (N=20)	DSL with SCS (N=28)	
Sensory Deficit	Present	4 (20%)	16 (57.1%)	0.01
	Absent	16 (80%)	12 (42.9%)	
Motor Power	Intact	18 (90%)	18 (64.3%)	0.042
	Reduced	2 (10%)	10 (35.7%)	
Straight Leg Raise Test	Positive	6 (30%)	12 (42.9%)	0.364
	Negative	14 (70%)	16 (57.1%)	
Deep Tendon Reflex	Intact	18 (90%)	12 (42.9%)	0.002
	Impaired	2 (10%)	16 (57.1%)	

Table 5 shows the relation of the specific MRI findings among the symptomatic patients suffering from DSL. Some of the key clinical presenting features have been assessed and compared with the facet joint angle at the level of the degenerative listhesis. Most of the symptomatic patients had facet joint angles more than 45 degrees at the level of the slip. However, there was no statically significant relation (p-value 0.767) of the presenting symptoms with the facet joint angle altogether.

**Table 5:** Clinical symptoms in patients with DSL and their facet joint angle at the level of deformity DSL: degenerative spondylolisthesis

Clinical Symptoms in patients with DSL	Facet Joint Angle		p-Value
	<45 Degree	>45 Degree	
Axial back pain only	8 (33.3%)	16 (66.7%)	0.767
Backache & extremity pain	8 (33.3%)	16 (66.7%)	
Claudication	8 (40%)	12 (60%)	
Radiculopathy	6 (25%)	18 (75%)	

## Discussion

DSL has been classically considered as a diagnostic subcategory in the generic diagnosis of SCS [7]. However, this is a separate entity among the degenerative lumbar spinal conditions with a different approach to its management [8]. Therefore, this study has tried to analyse the clinical presentation of DSL as an isolated entity and tried to discuss the impact of the concomitant presence of SCS on clinical symptomatology. This could give an estimate of how the clinical features are influenced by the presence of associated SCS. Like previous epidemiologic studies on the incidence of DSL, the incidence was higher among the female patients in the current study [9]. However, this study has focused more on the age and gender neutralised clinical presentation and findings among the symptomatic patients. Our study attempts to correlate the imaging findings of the condition. Classically, DSL is known to present with axial back pain, which is generally non-radiating in nature and located over the lower lumbar region. Many previous studies have considered segmental instability as the primary contributory factor for axial and paraspinal pain [10]. Often the grade of the spondylolisthesis is thought to contribute to the magnitude of the pain and rapidity of clinical presentation among the patients [11]. However, there are studies that have proven contradictory facts to this anecdotal conventional evidence. Pearson et al. [12] reported that there is no correlation between the incidence of back pain and the grading of the listhesis or the presence of instability on the dynamic flexion-extension radiograph. This is suggestive of the fact that imaging indicators of instability may not correlate well with clinical symptomatology [12].

On the other hand, the presence of canal stenosis with or without root encroachment can cause back pain with variable degrees of radiculopathy [10]. Therefore, associated canal stenosis can often modify the conventional symptoms of instability and the patients are more symptomatic because of the radicular pain itself. In a sense, true symptoms of instability can be very difficult to ‘pin-point’ and correlate with the radiographic estimation. However, in the current study, there has been a statistically significant incidence of axial back pain among the patients with radiological findings of DSL only on the MRI. Patients with features of SCS with DSL on the MRI had a higher incidence of radicular pain. However, this incidence of radiculopathy was not statistically significant. Therefore, it is practically very difficult to draw a clear line between the clinical presentation of axial back pain and radiculopathy purely based on the MRI findings. There have been some overlapping clinical features.

Interestingly, the incidence of neurogenic claudication was higher among the patients with MRI findings of associated SCS and this was statistically significant. In cases of degenerative lumbar spinal disease, different entities often co-exist. Therefore, the clinical features are variable with different possible conditions depending upon the predominant degenerative pathology [13]. The findings from our study support this idea. The clinical features can give the clinicians an indicative idea for diagnostic formulation to work-up on a group of differential diagnoses [3]. Degenerative lumbar spinal pathologies are complex and can result from the compounding effect of multiple co-existing factors. We feel that reconsideration of the clinical symptoms to be given once the clinician had the radiological diagnosis in place. This is crucial for targeted management.

In this study, we have tried to analyse the characteristics of distal extremity pain associated with back pain. Patients with canal stenosis had a higher incidence of pain radiating distal to the knee. Theoretically, this can be well explained by the MRI findings. However, looking into the clinical symptomatology first, this may be a difficult predicting factor as radicular pain had no statistically significant association with the imaging findings in our study. Ilyas et al. reported on a review of the Spine Patient-Reported Outcomes Related Trial (SPORT) literature expressing a similar view on the assessment of the DSL and lumbar spinal stenosis [14]. Although the SPORT study predominantly discussed treatment effects, clinical outcomes, and cost-effectiveness of the management of degenerative conditions, it also catered diagnostic information for clinical correlation.

Herron et al. [15] reported on a series of 140 surgically managed patients suffering from degenerative lumbar spinal pathologies with the predominant cohort affected by the SCS. Some of the patients in their series had DSL too. They noticed that improvement of the back pain and leg pain was not significantly associated with the presence or severity of listhesis. However, these findings are retrospective supportive evidence. Furthermore, there were no matching criteria used for the accompanying degenerative conditions. Therefore, it is difficult to isolate the causal effect of DSL, particularly, on the clinical features of back pain, radiculopathy, and claudication. This can be attributed to the natural course of the degeneration of the adult lumbar spine where changes affect intervertebral disc, vertebral column, facet joints, neural arch, and posterior ligamentous complex resulting in a heterogeneous condition [16]. Naturally, the clinical features and presentation are widely variable depending upon the predominant degenerative change. However, none of the conditions and the corresponding presentations are exclusive. Rather it is a diverse group of conditions, which need careful analysis of the symptoms, clinical findings, signs, and radiographic assessment (including gold standard investigation like MRI) to come to a balanced conclusion in order to formulate the management plan [17]. None of these factors can be a stand-alone decisive element. Instead, reconsideration of the clinical and imaging facts in light of each other would fabricate a safe meaningful outcome for the patients [18,19].

In the current study, patients with features of SCS in the MRI had a higher incidence of neurological deficit in at least one dermatome or myotome of the lower extremities. This was also statistically significant. Herkowitz et al. [20] have studied 50 cases of DSL with SCS for their surgical outcomes. Although this paper mostly discussed the surgical outcomes, they have also discussed the baseline presenting features for the patients. They have also noted statistically significant higher incidence of neurological deficit among patients with central canal and foraminal stenosis. Furthermore, they have also noted significant incidence of altered deep tendon reflexes in the lower limbs depending upon the severity of the canal stenosis. This is matching with our finding of statistically significant incidence of altered deep tendon reflexes in the lower extremities among the patients with a diagnosis of SCS together with DSL. Similarly, another study by Vaccaro et al. [21] noted a higher incidence of neurological findings among patients with SCS with DSL. Since they have compared the different surgical outcomes among their study population, they were able to establish the causal effect of the canal stenosis for neurological findings. In our study, we have primarily discussed the presenting clinical features. However, the trend for initial clinical presentation was quite similar to their analysis.

In our study, SLRT was mostly positive at 45 degrees or less among the patients of canal stenosis with degenerative listhesis. However, this association was not statistically significant. In the current study, we have noticed that clinical signs are significantly influenced by the radiographic presence of lumbar canal stenosis in the MRI. The only exception in our study was signs for sciatic nerve stretch as evidenced by the SLRT. Therefore, we can say that the predominant clinical features were dictated by the presence of lumbar canal stenosis in the MRI among the patients of degenerative lumbar spondylolisthesis. Radiographic instability of the lumbar spine in the flexion-extension radiograph with features of neurological deficit should warrant clinicians to investigate the condition further with MRI to isolate the surgical target. Undoubtedly, there has been crucial contribution of the correlation of the clinical and MRI findings for optimum management.

Cho et al. [22] have studied 54 cases of DSL with regards to the imaging findings. They have closely investigated all the cases with dynamic radiograph and MRI. Although MRI is the gold standard investigation for this condition, obtaining a plain radiograph may help in the diagnosis of anterior listhesis at the hypermobile segment, which may not be obvious on the MRI done in supine position. However, once the basic diagnosis of spondylolisthesis is obtained, MRI generally gives further information about the neural structures and associated degenerative conditions. Moreover, identification of fluid signal change in the facet joints on MRI is highly indicative of segmental instability [23]. Therefore, MRI has a notable impact as a radiographic diagnostic aid furnishing detailed information. In our study, we have measured the facet joint alignment at the level of instability. Patients with more than 45 degrees of facet joint angle were more symptomatic with respect to back pain, extremity pain, radiculopathy and claudication. However, this was not statistically significant. Eroglu et al. [24] have studied 612 facet angles in 53 patients comparing with 294 facet angles in 49 control subjects. They have found that facet joint tropism is significantly higher among the patients with spondylolysis and this could be a predisposing factor. However, this study was solely centred around spondylolytic spondylolisthesis. Therefore, the results of this study are not directly comparable to our study. At the time of writing this manuscript, there has been no literature evidence comparing the fact joint angle alignment with the clinical findings for DSL. Due to the unavailability of a literature benchmark, it is difficult to corroborate the association of facet joint alignment with the clinical symptomatology. 

We acknowledge the weakness of a single-centre study with a limited sample size. However, in the current literature, the evidence is limited on the clinical and imaging correlation with respect to DSL. Most of the available studies discuss primarily on the treatment and surgical outcomes. With all the limitations, we have tried to consider the impact of canal stenosis on the clinical presentation of the DSL with consideration of the MRI findings. Further prospective multicentre studies would help to build a better understanding of this topic. 

## Conclusions

DSL is a heterogeneous condition with the simultaneous presence of different degenerative processes in the lumbar spine at various stages. Hence, clinical presentations are widely variable. Concomitant presence of spinal canal stenosis significantly influences the clinical symptomatology with correlation to the MRI findings. Therefore, a judicious weighing of the clinical and imaging findings is crucial for prudent management planning for cases of DSL.
